# Changes in macrophage transcriptome associate with systemic sclerosis and mediate *GSDMA* contribution to disease risk

**DOI:** 10.1136/annrheumdis-2017-212454

**Published:** 2018-01-17

**Authors:** Aida Moreno-Moral, Marta Bagnati, Surya Koturan, Jeong-Hun Ko, Carmen Fonseca, Nathan Harmston, Laurence Game, Javier Martin, Voon Ong, David J Abraham, Christopher P Denton, Jacques Behmoaras, Enrico Petretto

**Affiliations:** 1 Centre for Computational Biology, Duke-NUS Medical School, Singapore, Singapore; 2 Centre for Complement and Inflammation Research, Hammersmith Hospital, Imperial College London, London, UK; 3 Division of Medicine, Department of Inflammation, Centre for Rheumatology and Connective Tissue Diseases, Royal Free and University College Medical School, University College London, London, UK; 4 Genomics Laboratory, MRC London Institute of Medical Sciences, Hammersmith Hospital, Imperial College London, London, UK; 5 Instituto de Parasitología y Biomedicina López Neyra, Consejo Superior de Investigaciones Cientıficas, Granada, Spain; 6 Faculty of Medicine, Medical Research Council (MRC) London Institute of Medical Sciences, Imperial College London, London, UK

**Keywords:** systemic sclerosis, macrophage, GSDMA, eQTL analysis

## Abstract

**Objectives:**

Several common and rare risk variants have been reported for systemic sclerosis (SSc), but the effector cell(s) mediating the function of these genetic variants remains to be elucidated. While innate immune cells have been proposed as the critical targets to interfere with the disease process underlying SSc, no studies have comprehensively established their effector role. Here we investigated the contribution of monocyte-derived macrophages (MDMs) in mediating genetic susceptibility to SSc.

**Methods:**

We carried out RNA sequencing and genome-wide genotyping in MDMs from 57 patients with SSc and 15 controls. Our differential expression and expression quantitative trait locus (eQTL) analysis in SSc was further integrated with epigenetic, expression and eQTL data from skin, monocytes, neutrophils and lymphocytes.

**Results:**

We identified 602 genes upregulated and downregulated in SSc macrophages that were significantly enriched for genes previously implicated in SSc susceptibility (P=5×10^−4^), and 270 *cis*-regulated genes in MDMs. Among these, *GSDMA* was reported to carry an SSc risk variant (rs3894194) regulating expression of neighbouring genes in blood. We show that *GSDMA* is upregulated in SSc MDMs (P=8.4×10^−4^) but not in the skin, and is a significant eQTL in SSc macrophages and lipopolysaccharide/interferon gamma (IFNγ)-stimulated monocytes. Furthermore, we identify an SSc macrophage transcriptome signature characterised by upregulation of glycolysis, hypoxia and mTOR signalling and a downregulation of IFNγ response pathways.

**Conclusions:**

Our data further establish the link between macrophages and SSc, and suggest that the contribution of the rs3894194 risk variant to SSc susceptibility can be mediated by *GSDMA* expression in macrophages.

## Introduction

Systemic sclerosis (SSc) is an intractable chronic autoimmune disease of unknown aetiology with high clinical heterogeneity and mortality rates. SSc is characterised by complex inflammatory, vascular and fibrogenic interactions occurring in multiple systems and tissues.^1^ Among the cellular populations contributing to the pathogenesis of SSc, monocytes/macrophages have been suggested to play a key role in initiating and/or perpetuating the disease,^2^ but their specific role and importance are still unclear. Candidate gene and genetic screen studies have begun to elucidate the genetic architecture of SSc^3^; for instance, genome-wide association studies (GWAS) and whole exome sequencing (WES)^4^ have reported numerous genes associated with susceptibility to SSc or to SSc subphenotypes and related traits.^5^ However the functional and cellular context of many genes and variants associated with SSc remains poorly understood. In macrophages, gene sets representative of macrophage activation have been used for enrichment analyses in expression profiles obtained from SSc-associated tissues,^6^ but the direct link between SSc disease variants and macrophage transcriptome remains to be elucidated.

Here we integrate differential expression and expression quantitative trait locus (eQTL) analyses in monocyte-derived macrophages (MDMs) from patients with SSc and healthy controls, revealing (1) changes in macrophage transcriptome as an important contributor to SSc and (2) upregulation and *cis*-regulation of *GSDMA* (a candidate gene for SSc susceptibility) contributing to disease risk in macrophages but not in skin.

## Methods

### Sample collection and clinical details

Patients with SSc met the American Rheumatism Association preliminary criteria for a diagnosis of SSc.^7^ The study was carried out with a total of 57 patients who attended the rheumatology clinic at the Royal Free Hospital exhibiting SSc with subgroups of limited cutaneous SSc (lcSSc) and diffuse cutaneous SSc (dcSSc). Patients with overlap features of another autoimmune rheumatic disease were excluded. Cases were classified as lcSSc or dcSSc according to extent of skin thickening^8^ and reflected the expected serological and clinical characteristics of the cohort that have been detailed in previous publications.^9^ Patients were receiving standard treatments for SSc in line with current European League Against Rheumatism recommendations.^10^ Thus, 11 patients were receiving low-dose prednisolone, 9 had received methotrexate, 18 received mycophenolate and 2 had received other potential disease-modifying agents (cyclophosphamide and rituximab, respectively). As expected, immunosuppression was more frequently used in cases with diffuse skin disease. Blood samples were also collected from 15 healthy control subjects. Details for all the samples included in this study can be found in online supplementary table S1. All subjects gave written informed consent. Blood (25 mL) was drawn from all patient and control samples using standardised phlebotomy procedures into sodium citrate tubes.

10.1136/annrheumdis-2017-212454.supp2 Supplementary file 2

### Isolation of MDMs

Human MDMs were differentiated from total blood from patients with SSc and healthy donors using gradient separation (Histopaque 1077, Sigma) and adhesion purification. Following Histopaque separation, peripheral blood mononuclear cells were resuspended in RPMI (Life Technologies), and monocytes were purified by adherence for 1 hour at 37°C, 5% CO_2_. The monolayer was washed three times with Hank’s Balance Salt Solution (HBSS) to remove non-adherent cells, and monocytes were matured for 5 days in RPMI containing 100 ng/mLmacrophage colony-stimulating factor (M-CSF) (PeproTech, London, UK) and 10% fetal calf serum (Labtech International). Macrophage purity was confirmed by immunohistochemical assessment of CD68 and >99% cells were CD68^+^.

### RNA extraction and RNA sequencing

Total RNA was extracted from human monocyte-derived macrophages (hMDMs) using TRIzol (Invitrogen) and RNeasy Mini Kit (Qiagen) according to manufacturers’ instructions, with an additional purification step by on-column DNase treatment using the RNase-Free DNase Kit (Qiagen) to ensure elimination of any genomic DNA. The integrity and quantity of total RNA were determined using a NanoDrop 1000 spectrophotometer (Thermo Fisher Scientific) and Agilent 2100 Bioanalyzer (Agilent Technologies). In total 500 ng of total RNA was used to generate RNA-sequencing (RNA-seq) libraries using TruSeq RNA Sample Preparation Kit (Illumina) according to the manufacturer’s instructions. Briefly, RNA was purified and fragmented using poly-T oligo-attached magnetic beads using two rounds of purification followed by the first and second complementary DNA (cDNA) strand synthesis. Next, cDNA 3' ends were adenylated and adapters ligated followed by 15 cycles of library amplification. Finally, the libraries were size-selected using AMPure XP Beads (Beckman Coulter), purified and their quality was checked using Agilent 2100 Bioanalyzer. Samples were randomised to avoid batch effects, and multiplexed libraries were run on a single lane (six samples/lane) of the HiSeq 2500 platform (Illumina) to generate 100 bp paired-end reads. An average coverage of 64M reads per sample was achieved. The RNA-seq data have been deposited in NCBI’s Gene Expression Omnibus (GEO) database (GEO Series accession number GSE104174).

### Quantitative reverse transcription PCR analysis

cDNA was obtained from 500 ng of total RNA using the Bio-Rad iScript Kit (Bio-Rad, Hertfordshire, UK) according to the manufacturer’s instructions. Quantitative reverse transcription PCR reactions were performed using the ViiA 7 Real-Time PCR System (Life Technologies). A total of 10 ng of cDNA per sample was used for PCR using Brilliant II SYBR Green qPCR Master Mix (Agilent Technologies). QuantStudio Real Time PCR Software (Life Technologies) was used for the determination of treshold cycle (Ct) values. Results were analysed using the comparative Ct method,^11^ and each sample was normalised to the reference gene (*HPRT*) to account for any cDNA loading differences. Results are expressed as mean±SEM, and statistical analysis was performed using Student’s t-test.

### Genotyping

DNA was isolated from 1 mL of whole blood of 71 samples (57 patients with SSc and 14 controls) using Gentra Puregene Blood Kit (Qiagen). Genotyping was performed on the Illumina Infinium Omni2.5–8 1.3 platform, which resulted in 2 372 784 genotype calls (Illumina GenomeStudio V.1.9.4 software).

RNA-seq and genotype data processing and detailed description of all the analyses included in this work can be found in the online supplementary file.

10.1136/annrheumdis-2017-212454.supp1 Supplementary file 1

## Results

### Differential expression analysis of SSc and control MDMs

**Figure 1:**
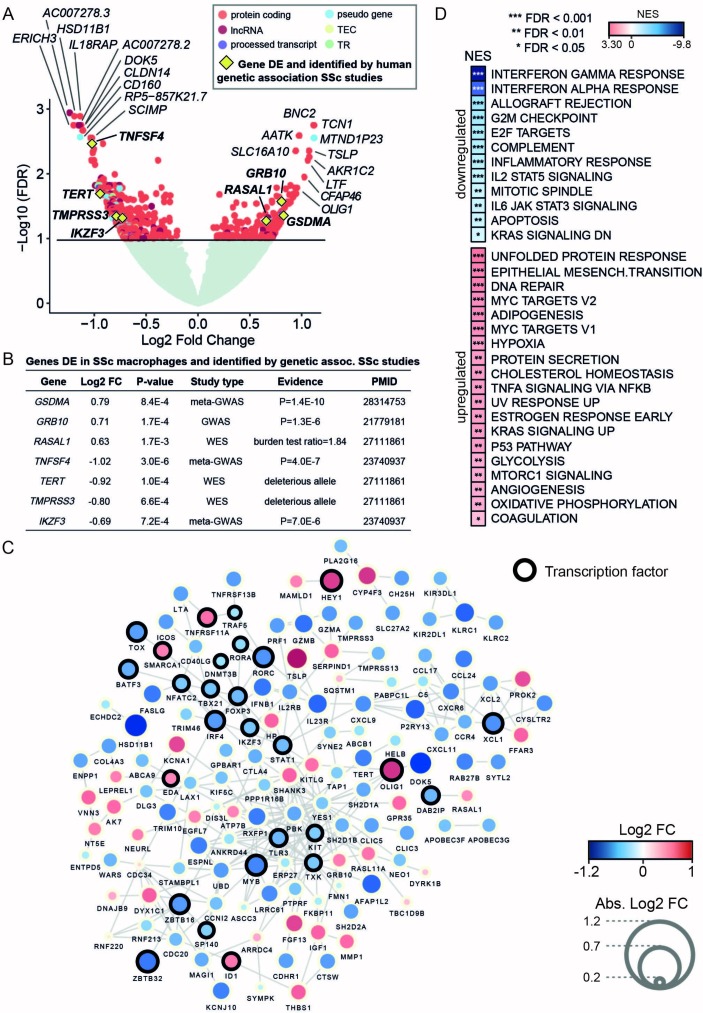
RNA sequencing differential expression analysis between monocyte-derived macrophages (MDMs) from patients with systemic sclerosis (SSc) and healthy controls provides evidence for the involvement of macrophages in SSc and related cellular processes. (A) Volcano plot with differential expression results. Gene names of the top 10 upregulated and downregulated genes are included. Genes previously identified in SSc human genetic association studies are also highlighted (yellow diamond). Genes with no significant differential expression are displayed in light green, whereas differentially expressed (DE) genes (false discovery rate (FDR) <0.1) are displayed coloured by gene type (TEC denotes gene to be experimentally confirmed and TR denotes T cell receptor genes). (B) Summary of the DE genes in SSc MDMs that have been previously found to be associated with SSc susceptibility by human genetic studies. The table includes differential expression test statistics in MDMs (log2 fold change (FC) and P value) and information about the SSc genetic study in which the gene had previously been reported (ie, study type and the provided evidence for involvement with SSc susceptibility). (C) Network with known protein-protein and databases interactions (edges) between the DE genes (nodes) identified in SSc MDMs. Gene size and colour are mapped to log2 FC. Only the genes with reported connections are displayed here (see Methods). (D) Functional processes (from Hallmark database) enriched in the set of DE genes in SSc MDMs were computed by gene set enrichment analysis (GSEA).^33^ Normalised enrichment scores (NES) denote the upregulation and downregulation enrichment strength. FDR levels for the GSEA are also included. GWAS, genome-wide association studies; WES, whole exome sequencing.

Differential expression analysis of MDMs expression profiles, in patients with SSc (n=57) and controls (n=15) (online supplementary figure S1), identified 170 upregulated and 432 downregulated genes in SSc, respectively (a total number of 602 genes, false discovery rate (FDR) <0.1; figure 1A and online supplementary table S2). Quantitative PCR analysis validated the changes detected by RNA-seq for a subgroup of genes (online supplementary figure S2). We revealed hundreds of genes associated with SSc in MDMs, including genes previously implicated in the genetic aetiology of the disease (figure 1B). In addition, 145 (25%) out of these 602 differentially expressed (DE) genes have been reported to interact functionally at the protein and pathway levels (figure 1C). Consistent with previously proposed biological processes and pathways associated with SSc,^3^ the upregulated genes showed significant enrichment for unfolded protein response, epithelial mesenchymal transition and DNA repair, whereas the downregulated genes showed enrichment for innate immune response-related processes (figure 1D), such as interferon response and allograft rejection, including genes previously linked to SSc (eg, *IL2RB*,^12^ *TNFAIP3*, *HLA-DQA1*, *HLA-DRB1* 3). Consistent with the previous SSc transcriptomics analysis in skin^13^ and in fibroblasts from patients with SSc-associated interstitial lung disease,^14^ the majority of the DE genes were downregulated in MDMs. The SSc macrophage transcriptome showed enrichment for genes involved in increased metabolic rates (glycolysis, hypoxia and mammalian target of rapamycin (mTOR) signalling), which have been previously linked with a proinflammatory activation profile.^15^

10.1136/annrheumdis-2017-212454.supp3 Supplementary file 3

To investigate whether the genes dysregulated in MDMs have been previously implicated with genetic susceptibility to SSc, we queried the National Human Genome Research Institute (NHGRI) GWAS Catalog^5^ and looked for genes identified by WES.^4^ We found that 10% of the genes previously associated with SSc overlapped with the set of DE genes detected in MDMs, representing a significant enrichment with respect to genome-wide expectation (10% observed overlap vs 2% expected overlap, Fisher’s exact test P=5×10^−4^; see online supplementary methods). Among the set of upregulated genes, we identified *GSDMA* and *GRB10*, which have previously been associated with SSc susceptibility^16^ and subphenotypes of SSc by GWAS,^17^ respectively. We also found *RASAL1*, a gene identified by WES that is enriched for deleterious variants in dcSSc.^4^ The set of downregulated genes included *IKZF3* and *TNFSF4*, both identified in a meta-GWAS SSc study,^18^ as well as *TERT* and *TMPRSS3*, candidate genes for dcSSc identified by WES.^4^ Therefore our DE analysis from patients with SSc and controls revealed hundreds of genes associated with SSc in MDMs, including genes previously implicated in the genetic aetiology of the disease.

### Genetic regulation of macrophage gene expression in SSc

**Figure 2:**
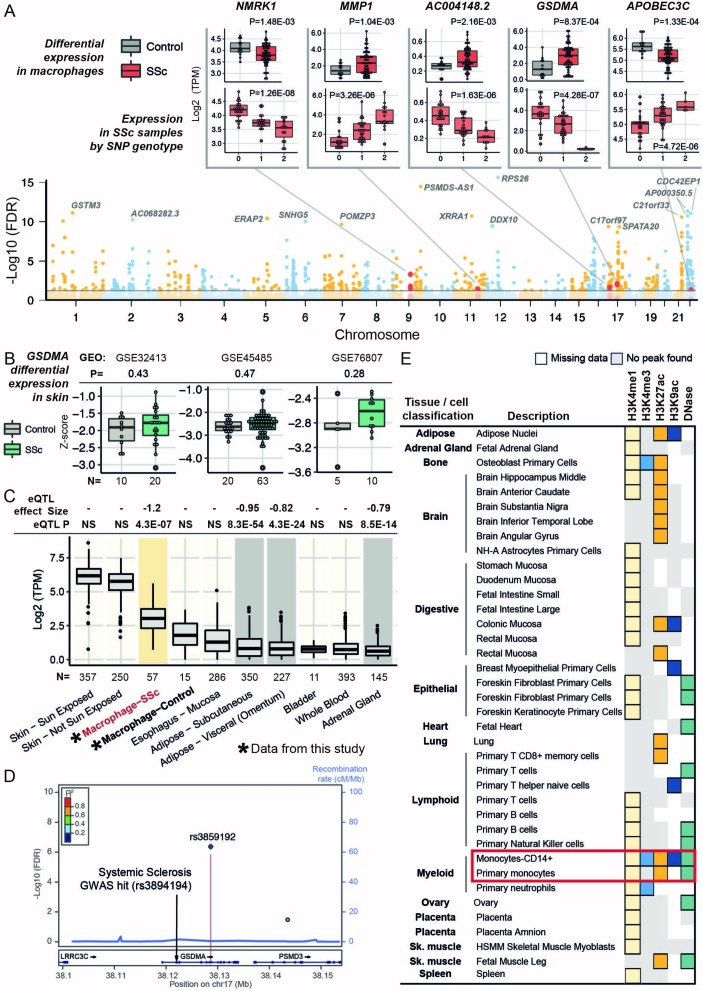
Study of *cis*-regulated genes in systemic sclerosis (SSc) monocyte-derived macrophages (MDMs). (A) Manhattan plot with all the *cis-*eQTL results. Differentially expressed genes in SSc MDMs (false discovery rate (FDR) <0.1) that are also *cis-*regulated (FDR <0.05) are highlighted in orange (five genes). Expression levels of these genes in SSc and control MDMs are displayed in boxplots (P refers to the P value of the differential expression test; see online supplementary methods). Expression levels of these five genes in patients with SSc according to the genotype of the *cis*-regulatory SNP (x-axis, the number refers to the number of copies for the minor allele) are shown in boxplots (P refers to the P value of the *cis*-eQTL; see online supplementary methods). (B) *GSDMA* expression levels in the skin in three cohorts of patients with SSc and controls (first two boxplots are cohorts of diffuse patients with SSc, whereas the third boxplot refers to patients with limited SSc). GEO refers to Gene Expression Omnibus database followed by the database accession number for each skin data set. P refers to t-test P value (two-tailed). N refers to the samples size in each group. (C) *GSDMA* expression levels in MDMs (from this study, indicated with asterisk) alongside with expression from all tissues/cell types included in Genotype-Tissue Expression (GTEx) database^24^ (only tissues/cell types with *GSDMA* median transcripts per kilobase million (TPM) levels >0.5 are displayed). We include at the top of the graph the tissues/cell types in which rs3859192 has been shown to regulate *GSDMA* levels in the GTEx database (both effect size and *cis*-eQTL P value are shown). Among the tissues where *GSDMA* is significantly *cis*-regulated (grey background), *GSDMA* is most highly expressed in SSc macrophages (highlighted with yellow background). In the case of the macrophage data, eQTL refers to the results presented in panel (A). (D) Overview of the genomic region on chromosome 17 centred on the *GSMDA* gene where we report the SNPs associated with *GSDMA* expression levels in macrophages (top eQTL SNP rs3859192). y-axis (left), significance of the eQTL in SSc macrophages is reported using FDR. y-axis (right), recombination rate between the SNPs. SNPs are displayed coloured by linkage disequilibrium (LD). Both LD and recombination rate are estimated from 1000 genomes (March 2012) in the European population. The location of the risk variant for SSc previously reported by Terao *et al* 16 (rs3894194) is also indicated (this SNP is not genotyped in the cohort used in this MDMs study). (E) Summary of the regulatory information (methylation, acetylation and DNase hypersensitivity) included in the Roadmap Epigenomics and ENCODE projects^27^ for the *cis*-eQTL SNP for *GSDMA* (rs3859192) detected in SSc macrophages (see online supplementary file for details). Red box, monocyte and monocyte-derived cell types. eQTL, expression quantitative trait locus; GWAS, genome-wide association studies; HSMM, human skeletal muscle myoblasts; SNP, single nucleotide polymorphism.

We carried out genome-wide *cis*-acting eQTL mapping in the cohort of patients with SSC (15 433 genes used as input; see online supplementary methods), which yielded 683 loci regulating the mRNA abundance of 270 genes in MDMs (genome-wide *cis*-eQTLs with FDR <5%; online supplementary table S2). The *cis*-regulated genes were nominally enriched for similar processes detected in the set of DE genes, such as interferon gamma (IFNγ) response and major histocompatibility complex class II protein complex (gene set enrichment analysis, P<0.02; online supplementary table S2). To identify *cis*-regulated genes associated with SSc, we integrated the eQTL data with the results of differential expression analysis (602 genes significantly differentially expressed with FDR <10%), which shortlisted five candidates, *GSDMA*, *MMP1*, *AC004148.2*, *APOBEC3C* and *NMRK1*, as the only genes that are both DE and *cis*-regulated in SSc MDMs (figure 2A). Among these candidates, *GSDMA* (Gasdermin A, a member of the Gsdm gene family that is required for tumour necrosis factor-α-induced apoptosis in mouse^19^) shows the highest upregulation in SSc MDMs. Furthermore, *GSDMA* is *cis*-regulated by the single nucleotide polymorphism (SNP) rs3859192 and is an established susceptibility gene for SSc (identified by the largest current transethnic meta-analysis comprising 4436 cases and 14 751 controls).^16^ Moreover, various genetic variants at the *GSDMA* locus have been previously associated with other autoimmune diseases with a proposed macrophage component, including asthma,^20^ rheumatoid arthritis,^21^ ulcerative colitis^22^ and Crohn’s disease.^23^ In contrast with the strong association of macrophage *GSDMA* expression with SSc reported here (P=8.4×10^−4^; figure 2A), *GSDMA* expression levels do not change in the skin of SSc (both diffuse and limited) in three independent studies (figure 2B). Despite *GSDMA* being most highly expressed in the skin as compared with 53 primary tissues/cell types analysed^24^ and the large sample size used for the *cis*-eQTL analysis (n≥250), the gene is not *cis*-regulated in the skin (normal and sun-exposed) (figure 2C).

To investigate whether the *cis*-regulation of *GSDMA* expression exists in a wider immune-cell context, we next assessed the genetic regulation of *GSDMA* expression in monocytes,^25^ neutrophils and lymphocytes.^26^ We did not find significant *cis*-regulation of *GSDMA* in basal (unstimulated) monocytes. Notably, the transcript was *cis*-regulated in monocytes stimulated with lipopolysaccharide at two time points (2 hours and 4 hours) and in IFNγ-stimulated (24 hours) monocytes^25^ (online supplementary table S4). These cis-regulatory SNPs found in stimulated monocytes included the *cis*-eQTL SNP rs3859192 found in MDMs from patients with SSc (figure 2D). No significant *cis*-regulation of *GSDMA* has been detected in neutrophils or lymphocytes.^26^ These results suggest that the functional relevance of *GSDMA* expression may be attributed to the monocyte/macrophage subset of the innate immune response.

10.1136/annrheumdis-2017-212454.supp5 Supplementary file 5

Our eQTL analysis revealed upregulation and significant *cis*-regulation of *GSDMA* mRNA levels in SSc MDMs (figure 2C), the latter is exerted by an intronic SNP rs3859192 (figure 2D). This eQTL SNP rs3859192 is in linkage disequilibrium (LD) with the risk variant rs3894194, which was found to be associated with SSc by transethnic meta-analysis.^16^ Specifically, the LD between rs3859192 and rs3894194 (estimated from 1000 genomes database) in European and African populations (represented in our multiethnic cohort of patients with SSc) is D'=0.72 (R^2^=0.49) and D'=0.92 (R^2^=0.66), respectively. We used Roadmap Epigenomics and ENCODE project data^27^ to search for additional evidence indicating a potential regulatory role of the *GSDMA* eQTL in MDMs (SNP rs3859192) and found, among other cell types, multiple overlapping regulatory marks in both primary and in CD14+ monocytes (figure 2E).

## Discussion

Large-scale genetic mapping studies have yielded novel hypotheses for genes and pathways associated with SSc.^3^ In addition to genetic studies, assessing the specific contribution of different cell types to the pathogenesis of SSc allows to decipher the functional context where disease susceptibility genes operate and eventually prioritise specific targets for therapeutic intervention. Host genetics influence the transcriptional response in human monocytes/macrophages in a cell-specific and stimulus-specific way and is associated with disease.^28^ Here we identified hundreds of genes whose expression level in macrophages is associated with SSc,^2^ highlighting a disease-mediating role for this cell type^2^. In comparison, a similar analysis between diffuse and limited SSc yielded only seven DE genes (online supplementary figure S3), suggesting that expression changes underlying clinical SSc subtypes might be more difficult to detect in macrophages.

Our results from differential expression and eQTL analysis in SSc macrophages, when combined with genetic susceptibility (GWAS/WES), regulatory (Roadmap Epigenomics and ENCODE) and expression and eQTL data from the skin and other cell types (GTEx^24^), support a previously undetected role for macrophages in *GSDMA* overexpression in the pathogenesis of SSc. In addition, the identification of a previously unappreciated macrophage *cis*-eQTL in LD with the previously reported SSc risk variant in *GSDMA* 16 suggests that the contribution to disease of *GSDMA* might be exerted by macrophages. *GSDMA* is a member of the recently discovered gasdermin protein family. Gasdermins were previously described as regulators of cellular swelling and lysis through formation of membranous pores in conjunction with release of proinflammatory cytokines, a process also known as pyroptosis.^29–31^ Accordingly, Gsdma3-mutant mice with constitutive pyroptosis display severe skin inflammation.^32^ Thus, we speculate that overexpression of *GSDMA* could cause dysregulation of the pyroptosis process in SSc. Taken together our integrated expression and eQTL analyses in SSc provide a proof of concept for the functional annotation of genes that have been implicated in disease susceptibility but are poorly characterised at the cellular level, prompting detailed functional studies of immune cells in SSc.

10.1136/annrheumdis-2017-212454.supp4 Supplementary file 4
