# Supplementary material for: Changes in macrophage transcriptome associate with systemic sclerosis and mediate GSDMA contribution to disease risk
Source: Ann Rheum Dis. 2018 Jan 17;77(4):596–601. doi: 10.1136/annrheumdis-2017-212454 (PMC5890626; doi:10.1136/annrheumdis-2017-212454)
Supplement: Supplementary file 1 [file annrheumdis-2017-212454supp001.docx]

**Changes in macrophage transcriptome associate with systemic sclerosis and mediate *GSDMA* contribution to disease risk**

Moreno-Moral^*^, Bagnati^*^ *et al*

**SUPPLEMENTARY FIGURES**

**Figure S1.** Heatmap with Z-score of adjusted Transcripts Per Kilobase Million (TPM) values of the significant differentially expressed (DE) genes (FDR<0.1, 602 genes) across all the samples included in this study when comparing SSc patients against control samples. Each row and column denote a DE gene and sample, respectively.

**Figure S2.** Quantitative real-time PCR validation of 10 selected differentially expressed genes (DE) on monocytes derived macrophages (MDM)s from controls (n=10) and SSc patients (n=10). Bars indicate mean ± SEM.

**
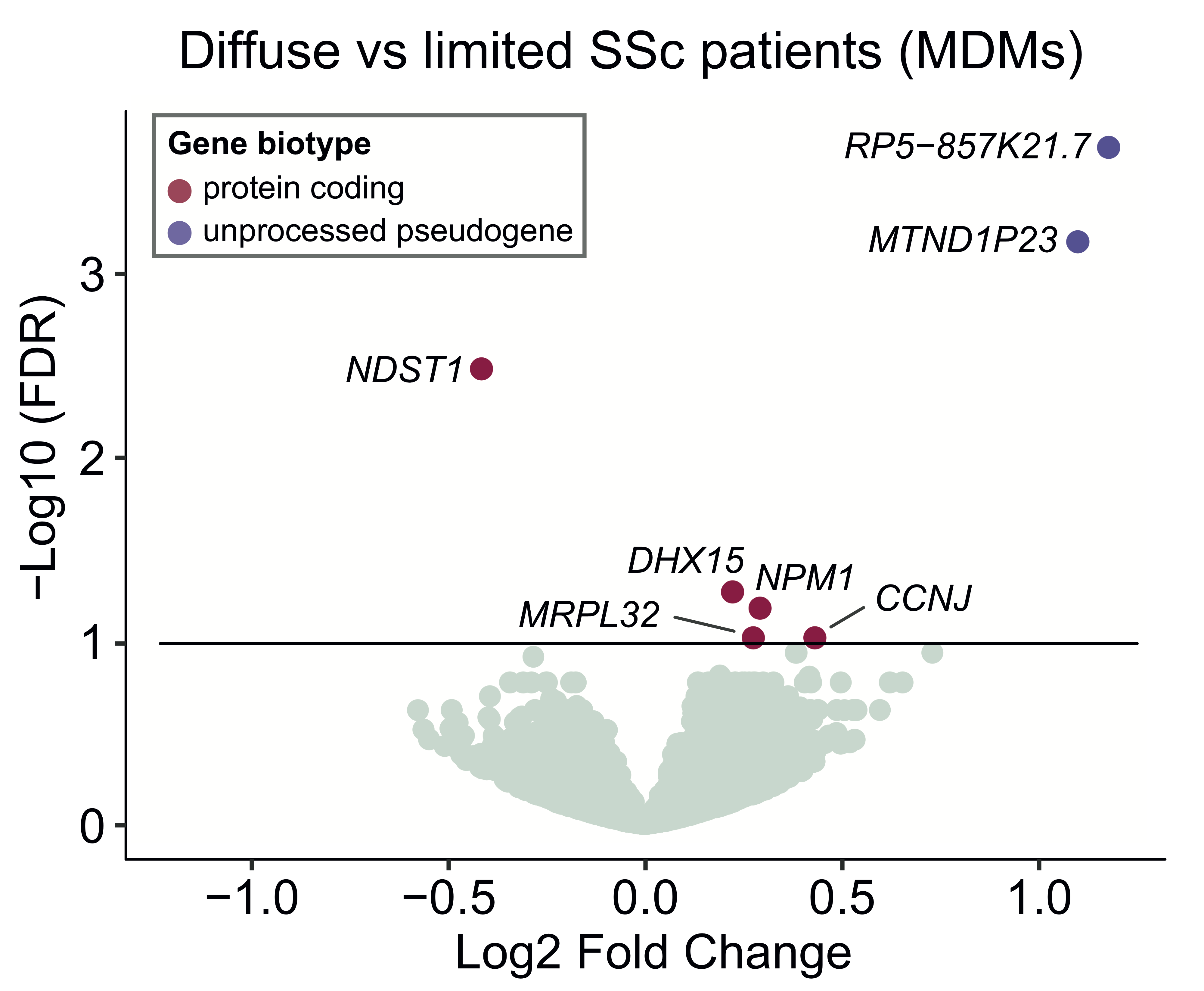
**

**Figure S3.** RNA-seq differential expression analysis between monocyte-derived macrophages (MDMs) from diffuse cutaneous systemic sclerosis (SSc) patients compared against MDMs from limited cutaneous SSc patients. Volcano plot with differential expression results. Genes with no significant differential expression are displayed in light green whereas significant differentially expressed (DE) genes (false discovery rate (FDR)<0.1) are displayed colored by gene biotype.

**Figure S4.** Quantile-quantile plot showing the distribution of the p-values of all cis-eQTL tests performed in the SSc patients (6,269,193 tests performed).

**SUPPLEMENTARY TABLES**

**Supplementary Table S1.** Patient data. Demographics and systemic sclerosis (SSc) subtype (limited or diffuse) of the samples included in this study. The treatments that the SSc patients were receiving are also included (empty field denotes patient not under treatment). NR (not reported). Ethnicity information C (caucasian), NC (non-caucasian).

**Supplementary Table S2.** RNA-seq differential expression (DE) analysis between monocyte-derived macrophages (MDMs) from systemic sclerosis (SSc) patients and healthy controls together with SSc MDMs *cis-*eQTL results. **Tab 1.** DE results with the top *cis-*regulatory SNP (SNP with the smallest FDR for each gene among all the *cis-*eQTL tests performed). Base Mean refers to the mean of *DESeq2* R package normalized counts in all samples. Log2 FC, Log2-fold changes computed by *DESeq2* package. DE FDR, Benjamini-Hochberg adjusted p-value (in this column “-“ denotes genes labelled as outlier by *DESeq2* Cook’s distance). In the *cis-*eQTL test columns, “-“ denotes genes not run in the *cis-*eQTL test (these did not pass the gene expression filtering step). **Tab 2.** List with all the genome-wide significant results (FDR<0.5) from the *cis-*eQTL test in the SSc MDMs cohort. **Tab 3.** Set composed of 73 genes reported in SSc genome-wide association studies (GWAS) and whole exome sequencing (WES). This gene set was used to test for enrichment of SSc genetic association studies-reported genes in the set of macrophage SSc DE genes. This list of genes was compiled as described in supplementary methods.

**Supplementary Table S3.** Functional gene set enrichment analysis (GSEA) results for the databases Hallmark, KEGG and GO (BP, CC and MP) in the RNA-seq differential expression (DE) analysis between monocyte-derived macrophages (MDMs) from systemic sclerosis (SSc) patients and healthy controls (tab 1), and *cis-*regulated genes in the SSc cohort (tab 2). GSEA was computed ranking all genes by *DESeq2* R package Wald test statistic. NES denotes normalized enrichment score. "P or N enrich" denotes whether the gene set is upregulated, P (positive), or downregulated, N (negative).

**Supplementary Table S4.** *GSDMA* is *cis-*regulated in activated monocytes in the study reported by Fairfax *et al* (Science 2014) by the same SNP that controls *GSMDA* expression levels in monocyte-derived macrophages (rs3859192). Neither rs3859192 nor rs3894194 have been reported as *cis-*regulatory SNPs for *GSDMA* in whole blood, lymphocytes or neutrophils in the study by Westra *et al* (Nature Genetics 2013). These results have been retrieved from Fairfax *et al*, Science 2014 (Supplementary Table S2) and Westra *et al*, Nature Genetics 2013 (Supplementary Table S3).

**SUPPLEMENTARY ONLINE METHODS**

**RNA-sequencing (RNA-seq) data processing**

RNA-seq reads were assessed for quality, aligned to hg38 (Ensembl Gene annotation build 79 using STAR 2.5.2b^1^) and quantified with RSEM 1.2.31^2^. The average mapping rate (unique and multimapping) was 93.97%. Gene annotation was retrieved from Ensembl version 79 (hg38) using the R library biomaRt 2.30.0^3^. Ribosomal genes (Ensembl gene biotype “rRNA”) and mitochondrial genes were removed (584 genes). Gene counts were rounded using the R function *round*^4^ and differential expression analysis was performed with DESeq2 1.14.1^5^ with a pre-filtering step in which we considered only genes with more than 1 count when summing up across all samples.

**Differential expression and pathway enrichment analysis**

DESeq2 was run pairwise comparing systemic sclerosis (SSc) against control macrophages using Wald test and default parameterizations. The differential expression test was also run in a second independent additional SSc subphenotype analysis in which we compared diffuse cutaneous SSc patients against limited cutaneous SSc patients (supplementary figure S3). In both pairwise tests, sex and lane were added as covariates in the model. Genes were considered significantly differentially expressed (DE) if Benjamini & Hochberg adjusted p-value (BH-adj.P or FDR) < 0.1 (see supplementary table S2 for full list of results SSc vs controls and Figure S3 for volcano graph with diffuse vs limited SSc comparison). DE genes resulted from the SSc vs control comparison were further analyzed and in following sections, DE genes will always refer to the set of DE genes from the SSc vs control pairwise test.

DE genes were tested for overrepresentation of SSc genome-wide association studies (GWAS) and whole exome sequencing (WES) reported genes by setting the background to the macrophages transcriptome (i.e. the input set of genes in the differential expression text). SSc GWAS reported genes were downloaded from NHGRI GWAS catalog^6^ (accessed on the 06/09/17), a total number of 41 unique genes. SSc (diffuse cutaneous, dcSSc) WES genes were downloaded from Mak *et al*^7^ (supplementary table 7 in the original manuscript; we downloaded the list of genes included in the groups “dcSSc only” and “dcSSc and ILD”), 32 unique genes. Among these 73 genes (41 GWAS and 32 WES genes, supplementary table S2), three genes (*SERPINB11*, *MUC7* and *SLC36A2*) did not pass the differential expression pre-filtering step (i.e. these genes were not expressed in the macrophage transcriptome). Therefore, 70 genes had been SSc associated (either GWAS or WES) and were expressed in macrophages. Out of these 70 genes, 7 were also DE in SSc macrophages, genes included in figure 1B (total number of DE genes: 602; input number of genes in the DE test (i.e. macrophage transcriptome): 30,254 genes). This contingency matrix was used to test for overrepresentation of SSc-associated genes in the set of macrophage DE genes by using a one-tailed Fisher’s exact text (*fisher.test* R function with *alternative* parameter set to *'greater'*).

Log2 of Transcripts Per Kilobase Million (TPM)+1 expression levels of the DE genes were adjusted for lane and sex effects by using the *removeBatchEffect* function from R library limma 3.30.13^8^, Z-scored and displayed in a heatmap (clustering performed using correlation distance and the method “ward.D” from *hclust* R function^4^, see supplementary figure S1). All DE genes can be found in supplementary table S1.

Functional enrichment analysis of the differential expression results was performed with Gene Set Enrichment Analysis (GSEA) software 2-2.2.2^9^. All genes included in DESeq2 output were mapped to HGNC symbols and ranked by the corresponding DESeq2 output Wald statistic (i.e. the estimate of the log2 fold change divided by its standard error). GSEA was run assessing overrepresentation of the following gene sets and pathways derived from the Molecular Signatures Database gene sets 5.1 (gene sets were queried using gene symbols): Hallmark gene sets (i.e. coherently expressed gene signatures derived from the aggregation of many MSigDB gene sets to represent well-defined biological states or processes), Gene Ontology (GO) and Kyoto Encyclopedia of Genes (KEGG) pathways. GSEA was run in classic pre-rank mode with 10,000 permutations to assess the false discovery rate (FDR). In the GSEA runs, maximum gene set size was set to 5,000 and minimum cluster size was set to 10. Gene sets were considered enriched if FDR<0.05. All Hallmark enriched gene sets are displayed in figure 1D. The rest of the results can be found in supplementary table S2.

STRING protein-protein interaction database 10.0^10^ was queried with the list of significantly DE genes (FDR<0.1) on the 28/08/2017 by using Ensembl gene IDs. The resulting network (composed by all experimental and databases connections with a minimum interaction score of 0.1) was retrieved and imported into Cytoscape^11^ to generate a network visualization (see Figure 1C). In the network graph, node size was mapped to absolute differential expression log2 fold changes, node color was mapped to the log2 fold change direction and genes annotated as transcription factor were highlighted.

**Quality control (QC) of the genotype data**

QC was performed using PLINK 1.9^12^. In more detail, we checked for sex mismatches, related individuals and removed SNPs with Hardy-Weinberg equilibrium exact test p-value smaller than 0.001 (--hwe 0.001, default value in PLINK 1.7). We also removed SNPs with a missingness per marker >5% and a missingness per individual of >3% (--geno 0.05 and --mind 0.03, respectively, cutoffs chosen based on the distribution of the obtained cumulative non-missingness distributions). The minimum allele frequency was set to 0.1 (--maf 0.1). All the samples and 1,056,502 SNPs passed these QC steps. An additional variant 0.8 r^2^ LD pruning step was also carried out with PLINK by using the --indep-pairphase option (i.e. r^2^ values are based on maximum likelihood phasing instead of correlation) with a window size of 2000 and a step size of 200. We removed SNPs with no chromosomal annotation and mitochondrial SNPs. All of this resulted in a final number of 577,402 SNPs to be used for *cis-*eQTL analysis.

***Cis-*eQTL analysis**

Log2 (TPM+1) gene expression was used in the *cis-*eQTL analysis after a gene expression filtering step carried out considering all SSc and controls samples. We classified all Ensembl gene biotypes into 10 broader gene categories: protein coding, pseudogenes (gene biotypes including “pseudo” in the category name) IG (gene biotypes containing “IG” in the category name without considering pseudogenes), TR (gene biotypes including “TR” in the category name without considering pseudogenes), lncrna ("3prime overlapping ncrna", "antisense", "known ncrna", "sense intronic", "lincRNA", "sense overlapping" and "macro lncRNA"), snrna (including "misc RNA", "snRNA", "miRNA", "snoRNA", "rRNA", "Mt tRNA", "Mt rRNA", "vaultRNA", "scaRNA" and "sRNA"), processed transcript, non-coding, ribozyme and TEC. Due to the diverse expression profile of all these 10 genes categories, we carried out an independent gene pre-filtering step in each of these gene categories. We removed the lower 10^th^ percentile of the median gene expression distribution (without considering genes with median gene expression of 0). In the case of the protein coding genes, we only kept genes with a median TPM expression levels higher than 1. Gene annotation in hg19 was downloaded using R library biomaRt (Ensembl Gene annotation build 75). Only genes that passed the filtering step and were included this Ensembl Gene annotation were considered for *cis*-eQTL analysis (total number of 15,433 genes).

Matrix eQTL (version 2.1.1) was used to compute *cis-*eQTL in the SSc cohort and healthy controls separately. We tested all *cis-*regulatory SNPs located within 1Mb of the gene by using the hg19 genome (Ensembl Gene annotation build 75) and a linear model. To account for covariates effect and population stratification, we included lane, sex and the first 5 principal components of the genotype data computed with PLINK in the 0.8 LD-filtered data in SSc and control cohorts respectively. *Cis-*eQTLs were deemed significant if FDR<0.05. The distribution of the p-values obtained in all performed tests is shown in supplementary figure S2. The top *cis-*associated SNP with each gene in the SSc cohort (SNP with the smallest FDR) can be found in supplementary table S1. Expression levels of genes (log2 TPM after adjusting for lane and sex as described above) that were DE (FDR < 0.1) and *cis-*regulated (FDR < 0.05) are displayed in boxplots (figure 2A).

The *cis-*eQTL p-value of all the SNPs associated with *GSDMA* gene (nominal p-value < 0.05) was input into Locuszoom 1.3^13^ to generate a visualization of the local association centered around *GSDMA* (figure 1D). Locus zoom was ran using the hg19 genome built with the linkage disequilibrium computed from 1000 Genomes (March 2012) in the European population. We queried the pairwise LD between rs3859192 and SSc associated SNP located within *GSDMA* reported by Terao et al.^14^ in different populations from 1000 genomes using LDassoc tool^15^.

We assessed the number of tissues/cell types in which rs3859192 regulates GSDMA by querying Genotype-Tissue Expression (GTEx) portal^16^ on the 9/09/17, these results are shown in figure 2C. Non-coding annotation of the top SNP regulating *GSDMA* levels in *cis* (rs3859192) were retrieved from HaploReg 4.1^17^ (figure 2D).

**Analysis of *GSDMA* expression profile**

GTEx project RNA-seq V6p gene read counts, reference annotation file and sample attributes were downloaded. TPMs were computed by using the provided gene reference annotation file. The samples (n=8555) were classified by tissue type as included in the column “SMTSD” of the sample attributes file (53 tissues). TPM of the SSc and control macrophage RNA-seq data generated in this study was added to this GTEx data and all the samples were quantile normalized together by using the function *normalize.quantiles* from the preprocessCore 1.36.0 R library^18^. Boxplots of *GSDMA* levels in all the tissues with a median TPM level higher than 0.5 can be seen in figure 2B.

*GSE76807*, *GSE45485* and *GSE76807* SSc and control normalized skin expression data were retrieved from Gene Expression Omnibus (GEO) database by using the R function *getGEO* from GEOquery R package^19^. By using the provided phenotypic information, all the SSc (only SSc with no treatment were considered) and control samples were selected. The intensity levels (Z-scored) of the probe corresponding to the *GSMDA* gene (as in the provided gene annotation), were represented in a boxplot in each dataset. In each boxplot, a two-sided t-test was computed in R^4^ to assess differences in *GSDMA* levels between in SSc and control skin samples.

**REFERENCES**

1. Dobin A, Davis CA, Schlesinger F, et al. STAR: ultrafast universal RNA-seq aligner. *Bioinformatics* 2013; 29: 15–21.

2. Li B, Dewey CN. RSEM: accurate transcript quantification from RNA-Seq data with or without a reference genome. *BMC Bioinformatics* 2011; 12: 323.

3. Durinck S, Spellman PT, Birney E, et al. Mapping identifiers for the integration of genomic datasets with the R/Bioconductor package biomaRt. *Nat Protoc* 2009; 4: 1184–1191.

4. R Core Team. R: A Language and Environment for Statistical Computinghttps://www.r-project.org/ (2016).

5. Love MI, Huber W, Anders S. Moderated estimation of fold change and dispersion for RNA-seq data with DESeq2. *Genome Biol* 2014; 15: 550.

6. MacArthur J, Bowler E, Cerezo M, et al. The new NHGRI-EBI Catalog of published genome-wide association studies (GWAS Catalog). *Nucleic Acids Res* 2017; 45: D896–D901.

7. Mak ACY, Tang PLF, Cleveland C, et al. Brief Report: Whole-Exome Sequencing for Identification of Potential Causal Variants for Diffuse Cutaneous Systemic Sclerosis. *Arthritis Rheumatol (Hoboken, NJ)* 2016; 68: 2257–62.

8. Ritchie ME, Phipson B, Wu D, et al. limma powers differential expression analyses for RNA-sequencing and microarray studies. *Nucleic Acids Res* 2015; 43: e47–e47.

9. Subramanian A, Tamayo P, Mootha VK, et al. Gene set enrichment analysis: a knowledge-based approach for interpreting genome-wide expression profiles. *Proc Natl Acad Sci U S A* 2005; 102: 15545–50.

10. Szklarczyk D, Franceschini A, Wyder S, et al. STRING v10: protein-protein interaction networks, integrated over the tree of life. *Nucleic Acids Res* 2015; 43: D447-52.

11. Shannon P. Cytoscape: A Software Environment for Integrated Models of Biomolecular Interaction Networks. *Genome Res* 2003; 13: 2498–2504.

12. Sambo F, Di Camillo B, Toffolo G, et al. Compression and fast retrieval of SNP data. *Bioinformatics* 2014; 30: 3078–3085.

13. Pruim RJ, Welch RP, Sanna S, et al. LocusZoom: regional visualization of genome-wide association scan results. *Bioinformatics* 2010; 26: 2336–2337.

14. Terao C, Kawaguchi T, Dieude P, et al. Transethnic meta-analysis identifies GSDMA and PRDM1 as susceptibility genes to systemic sclerosis. *Ann Rheum Dis* 2017; 76: 1150–1158.

15. Machiela MJ, Chanock SJ. LDassoc: an online tool for interactively exploring genome-wide association study results and prioritizing variants for functional investigation. *Bioinformatics*. Epub ahead of print 12 September 2017. DOI: 10.1093/bioinformatics/btx561.

16. Ardlie KG, Deluca DS, Segre A V., et al. The Genotype-Tissue Expression (GTEx) pilot analysis: Multitissue gene regulation in humans. *Science (80- )* 2015; 348: 648–660.

17. Ward LD, Kellis M. HaploReg: a resource for exploring chromatin states, conservation, and regulatory motif alterations within sets of genetically linked variants. *Nucleic Acids Res* 2012; 40: D930–D934.

18. Bolstad BM. preprocessCore: A collection of pre-processing functionshttps://github.com/bmbolstad/preprocessCore (2016).

19. Davis S, Meltzer PS. GEOquery: a bridge between the Gene Expression Omnibus (GEO) and BioConductor. *Bioinformatics* 2007; 23: 1846–1847.
